# Host plant species determines symbiotic bacterial community mediating suppression of plant defenses

**DOI:** 10.1038/srep39690

**Published:** 2017-01-03

**Authors:** Seung Ho Chung, Erin D. Scully, Michelle Peiffer, Scott M. Geib, Cristina Rosa, Kelli Hoover, Gary W. Felton

**Affiliations:** 1 Department of Entomology, Cornell University, Ithaca, NY 14853, USA; 2 Stored Product Insect and Engineering Research Unit, USDA-Agricultural Research Service, Center for Grain and Animal Health Research, Manhattan, KS 66502, USA; 3 Department of Entomology, Pennsylvania State University, University Park, PA 16802, USA; 4 Tropical Crop and Commodity Protection Research Unit, USDA-ARS Daniel K. Inouye Pacific Basin Agricultural Research Center, Hilo, HI 96720, USA; 5 Department of Plant Pathology and Environmental Microbiology, Pennsylvania State University, University Park, PA 16802, USA

## Abstract

Herbivore associated bacteria are vital mediators of plant and insect interactions. Host plants play an important role in shaping the gut bacterial community of insects. Colorado potato beetles (CPB; *Leptinotarsa decemlineata*) use several *Solanum* plants as hosts in their natural environment. We previously showed that symbiotic gut bacteria from CPB larvae suppressed jasmonate (JA)-induced defenses in tomato. However, little is known about how changes in the bacterial community may be involved in the manipulation of induced defenses in wild and cultivated *Solanum* plants of CPB. Here, we examined suppression of JA-mediated defense in wild and cultivated hosts of CPB by chemical elicitors and their symbiotic bacteria. Furthermore, we investigated associations between the gut bacterial community and suppression of plant defenses using 16 S rRNA amplicon sequencing. Symbiotic bacteria decreased plant defenses in all *Solanum* hosts and there were different gut bacterial communities in CPB fed on different host plants. When larvae were reared on different hosts, defense suppression differed among host plants. These results demonstrate that host plants influence herbivore gut bacterial communities and consequently affect the herbivore’s ability to manipulate JA-mediated plant defenses. Thus, the presence of symbiotic bacteria that suppress plant defenses might help CPB adapt to host plants.

As sessile organisms, plants are attacked by pathogens and insect herbivores and have developed strategies (induced and constitutive defense responses) to ward off these threats. Plant hormones such as jasmonic acid (JA), ethylene and salicylic acid (SA) regulate induced defenses^1^. In general, crosstalk between JA and SA plays a central role in modulating defense signaling networks^2^. The feeding mode of herbivores determines the timing, intensity, and composition of plant hormones that trigger appropriate defense signaling pathways. The JA-regulated pathway is often induced by chewing herbivores/necrotrophic pathogens, but the SA-responsive defense pathway is generally induced by herbivores with piercing-sucking mouthparts and biotrophic pathogens^3^.

Some herbivores exploit the antagonistic interactions between JA and SA to overcome induced host plant defenses by simultaneously triggering both signaling pathways. It has been shown that insect-derived effectors in oral secretions (OS) such as saliva and/or regurgitant manipulate plant defenses^4^. For example, silverleaf whiteflies (*Bemisia tabaci*) induce the SA-signaling pathway, which suppresses JA-regulated defenses in Arabidopsis, benefitting insect performance^5^. Beet armyworm caterpillars (*Spodoptera exigua*) suppress JA-dependent defenses by activating SA-responsive pathways^6^. In general, negative crosstalk between JA and SA is considered to occur in most plants^7^ and, therefore, it is not surprising that many insect species manipulate the interactions between these pathways to overcome induced plant defenses.

We previously demonstrated that symbiotic bacteria in OS from Colorado potato beetle larvae (CPB; *Leptinotarsa decemlineata*) suppressed JA-induced plant defenses^8^,^9^. These bacteria were released from OS when insects fed on tomato leaves and triggered the plant to induce the SA-signaling pathway, which, in turn, suppressed JA-regulated anti-herbivore defenses. In wild and cultivated *Solanum* species, JA is known to induce defensive secondary metabolites and proteins^10^,^11^,^12^. It is likely that JA-suppression by symbiotic bacteria in OS of CPB directly prevents accumulation of these defensive compounds.

CPB is a major pest of Solanaceous crops, such as potato (*Solanum tuberosum*), tomato (*S. lycopersicum*), and eggplant (*S. melongena*)^13^. CPB originated in southern Mexico where the major host plants are buffalobur (*Solanum rostratum*) and *S.angustifolium*^14^. It was believed that the expansion of CBP from the southwestern US to the eastern US was facilitated by a host range expansion that includes potatoes^15^. CPB can colonize a diverse range of host plants, but their primary hosts varies based on geographic location^16^,^17^. For example, in the Southwestern U.S., buffalobur and silverleaf nightshade (*S. eleaegnifolium*) are major hosts. In the central and southeastern US, horsenettle is a prevalent host^14^. Bittersweet nightshade (*S. dulcamara*, hereafter referred to as nightshade), which is prominent in the Northeastern U.S., can also support CPB populations^18^.

Several studies have described gut bacterial communities of herbivores and demonstrated that these bacteria play important roles in interactions with both the herbivore’s host plants and herbivore performance. Herbivore associated bacteria can provision nutrients to their insect hosts and protect against natural enemies and thermal stress^19^. In addition, microbial partners help insects detoxify plant toxins and adapt to specific host plants^20^,^21^,^22^. The composition and structure of the microbial community harbored by insects can be shaped by diet, developmental stages, geographic location, and physiochemical conditions^23^. Bacterial communities can vary depending on diet in several insect species^24^,^25^,^26^,^27^,^28^. However, few studies have determined how these shifts in bacterial community composition impact interactions between insects and their host plants or how these changes impact induced plant defenses.

In this study, we hypothesized that the gut symbiotic bacteria of CPB varies by host plant and that the symbionts differentially suppress induced defenses in wild and cultivated *Solanum* host plants. To test this hypothesis, we investigated whether 1) antagonistic interactions between JA and SA are present in *Solanum* hosts; 2) the symbiotic bacteria of CPB larvae inhibit defenses in a variety of hosts of CPB; and 3) host plant identity affects the composition and structure of CPB bacterial communities and the relative abundance of defense-suppressing bacteria. Overall, we found that host plants shape the diversity and abundance of gut symbiotic bacteria and this variation in the microbiota influences the degree to which plant defenses are suppressed in *Solanum* hosts.

## Results

### Negative crosstalk between JA and SA occurs in *Solanum* hosts

To investigate whether wild and cultivated *Solanum* plants displayed antagonistic interactions between JA and SA, we sprayed MeJA, SA or both chemical elicitors on plants and measured PPO activity 48 h after treatment. Overall, MeJA induced PPO activity in all plants, but the PPO activity levels in plants sprayed with SA were similar to control plants sprayed with EtOH (Supplementary Fig. S1). The application of both chemicals on tomato decreased PPO activity compared to plants treated with MeJA alone; however, PPO activity levels were still elevated compared to plants treated with either EtOH or SA. In the five other plant species, application of both elicitors decreased PPO activity compared to those treated with MeJA and the activity levels were similar to plants treated with EtOH or SA (tomato, *F*_(3,16)_ = 87.21, *P* < 0.0001; potato, *F*_(3,20)_ = 41.92, *P* < 0.0001; eggplant, *F*_(3,20)_ = 3.99, *P = *0.0223; buffalobur, *F*_(3,112)_ = 13.47, *P* < 0.0001; horsenettle, *F*_(3,19)_ = 4.31, *P* = 0.0177; nightshade, *F*_(3,34)_ = 4.18, *P* = 0.0127).

In addition to defensive protein activity, we determined if negative crosstalk between JA and SA impacted induced resistance of the six plant species to CPB larvae. Neonate larvae were fed on excised leaves from plants that were sprayed with MeJA, SA or both. Larval mass, which was used as a proxy for neonate performance, was negatively correlated with PPO activity levels (Supplementary Fig. S2). Larval growth on MeJA-treated plants of all six plant species was significantly lower than larval growth on SA- or EtOH- treated plants. Larval growth on tomato or horsenettle treated with both SA and JA was higher compared to larvae reared on MeJA-treated plants, but growth was less for CPB larvae reared on EtOH or SA-treated plants. On the four other plant species, larval growth on plants treated with both JA and SA was similar to larval growth on plants treated with EtOH or SA (tomato, *F*_(3,52)_ = 8.55, *P = *0.0001; potato, *F*_(3,107)_ = 3.27, *P* = 0.0240; eggplant, *F*_(3,86)_ = 5.93, *P = *0.0010; buffalobur, *F*_(3,110)_ = 7.10, *P* = 0.0002; horsenettle, *F*_(3,19)_ = 4.31, *P* = 0.0177; nightshade, *F*_(3,112)_ = 5.07, *P* = 0.0025).

**Figure 1:**
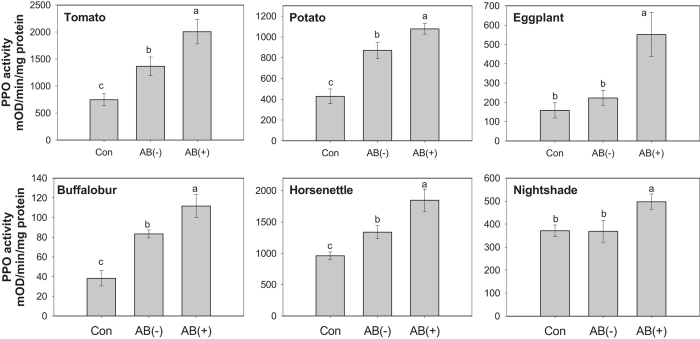
PPO activities in six different host plants damaged by AB-treated or untreated larvae of Colorado potato beetle reared on tomato. PPO activity was measured 48 h after insect feeding. Values are means ± SEM. Different letters above the bars represent significant differences (ANOVA, *P* < 0.05, *N* = 5–8). Con, undamaged plants; AB (−), plants damaged by untreated larvae; AB ( + ), plants damaged by AB-treated larvae.

We previously demonstrated that symbiotic bacteria in CPB larval OS manipulated JA-induced defenses in tomato^9^. Thus, to investigate whether symbiotic bacteria from CPB larvae reared on tomato suppresses plant defenses in other *Solanum* plants, plants were damaged by larvae that fed on either AB-treated leaves or untreated leaves. PPO activity was measured 48 h after insect introduction. In all *Solanum* plants, untreated larvae decreased PPO activity compared to AB-treated larvae (Fig. 1; tomato, *F*_(2,8)_ = 10.12, *P* = 0.0056; potato, *F*_(2,16)_ = 21.90, *P* < 0.0001; eggplant, *F*_(2,12)_ = 8.30, *P* = 0.0055; buffalobur, *F*_(2,18)_ = 19.34, *P* < 0.0001; horsenettle, *F*_(2,14)_ = 22.12, *P* < 0.0001; nightshade, *F*_(2,19)_ = 3.73, *P* = 0.0431).

### Specificity of defense suppression by larvae reared on *Solanum* hosts

**Figure 2:**
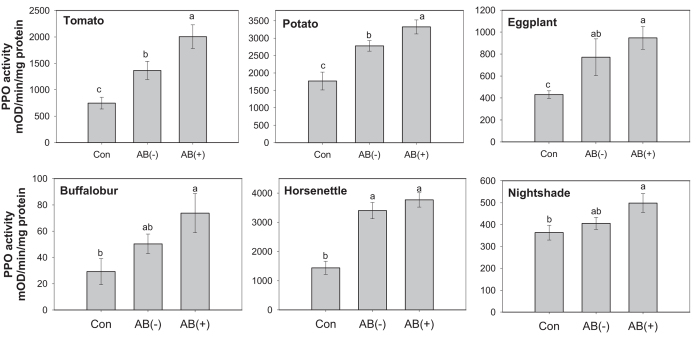
PPO activities in six different host plants damaged by AB-treated or untreated larvae of Colorado potato beetle reared on the corresponding host plants until third instar. PPO activity was measured 48 h after insect feeding. Values are means ± SEM. Different letters above the bars represent significant differences (ANOVA, *P* < 0.05, *N* = 5–10). Con, undamaged plants; AB (−), plants damaged by untreated larvae; AB (+), plants damaged by AB-treated larvae.

CPB larvae can successfully use wild and cultivated *Solanum* plants in their natural environment. Thus, to investigate whether symbiotic bacteria from larvae that were reared on non-tomato hosts suppress plant defenses in their corresponding plants, neonates from the tomato reared lab colony were placed to feed on one of five other hosts until they reached the third instar. AB-untreated larvae that fed on tomato and potato decreased PPO activity in tomato and potato, respectively, compared to AB-treated larvae (Fig. 2; tomato, *F*_(2,8)_ = 31.28, *P* = 0.0002; potato, *F*_(2,24)_ = 5.47, *P* = 0.0110). In contrast, when larvae were reared on other host plants, those larvae did not suppress PPO activity in these plants.

### Differential amounts of defense-suppressing bacteria and oral secretions by larvae reared on *Solanum* hosts

**Figure 3:**
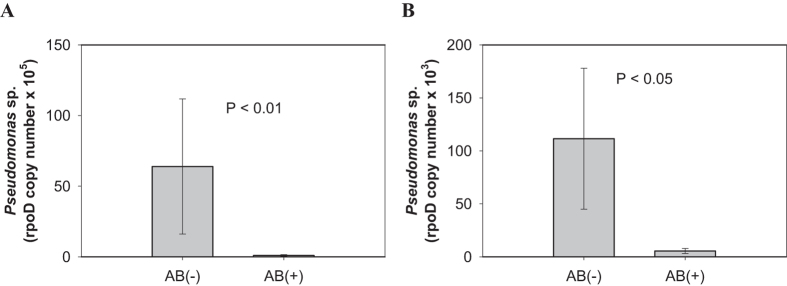
(**A**) Amount of *Pseudomonas* sp. deposited on potato leaves by Colorado potato beetle larvae reared on tomato represented by *rpoD* gene copy number. (**B**) The amount of *Pseudomonas* sp. deposited on potato leaves by larvae reared on potato using the same gene. *rpoD* copy numbers were measured 2 h after insect feeding. Values are untransformed means ± SEM (*N* = 5). AB (−), plants damaged by untreated larvae; AB (+), plants damaged by AB-treated larvae.

To investigate whether defense-suppressing bacteria are secreted by larvae that were reared on different hosts, we measured abundance of the defense-suppressing bacteria *Pseudomonas* sp. that were deposited on leaves during larval feeding using a specific primer for the *rpoD* gene. When tomato-fed larvae were placed on potato, AB-untreated larvae secreted more *Pseudomonas* sp. compared to AB-treated larvae (Fig. 3A; *t*_(8)_ = 3.93, *P* = 0.0004). When potato-fed larvae were placed on potato, a similar pattern was observed (Fig. 3B; *t*_(8)_ = 2.41, *P* = 0.0424). We could not detect *rpoD* on undamaged plants (Supplementary Table S1). Furthermore, *Pseudomonas* sp. was not detected on buffalobur by larvae that were reared on buffalobur (Supplementary Table S1).

To investigate whether host plants affect the amount of OS secretions deposited on their corresponding hosts, we quantified the intensity of a fluorescent dye on the wounded areas of leaves. Potato-fed larvae secreted more OS on potato than tomato-fed larvae did on tomato (Supplementary Fig. S3; *t*_(15)_ = −3.26, *P* = 0.0001). In contrast, eggplant- and horsenettle-fed larvae secreted less OS on eggplant and horsenettle, respectively (eggplant; *t*_(14)_ = 3.72, *P* = 0.0022; horsenettle; *t*_(14)_ = 4.49, *P* = 0.0005).

### Changes in bacterial communities of larvae reared on *Solanum* hosts

To assess the effect of host plants on gut microbial community structure and composition of CPB larvae, 16 S rRNA amplicon sequencing was conducted. The majority of the rarefaction curves approached saturation, indicating that our sequencing depth was sufficient to detect the majority of the abundant operational taxonomic units (OTUs) associated with each community (Supplementary Fig. S4). The observed richness of the CPB gut bacterial communities, indicated by the number of observed OTUs, varied among individuals feeding on different hosts (Table 1). For example, richness was highest in communities associated with beetles fed on tomato (69.0 ± 5.9 OTUs), while richness was considerably lower for larvae fed on horsenettle (15.7 ± 0.7 OTUs) and eggplant (30.7 ± 8.0 OTUs) compared to the four other plant species. Likewise, the Chao 1 richness values of gut bacterial communities in larvae fed on horsenettle and eggplant were the lowest, indicating that these communities had lower numbers of rare OTUs compared to communities associated with CPB larva fed on other plants. Diversity indices were highest for beetles fed on tomato plants, as indicated by the Shannon diversity index (1.85 ± 0.52). However, there were no significant differences in richness (Chao 1) and diversity indexes (Shannon) between tomato-fed larvae and other samples due to the high variation among biological replicates within treatment (Wilcoxon rank test, P > 0.05). Without removing singletons, the number of observed OTUs and Chao 1 richness values increased but overall pattern of alpha diversity indexes did not change (Supplementary Table S2).

**Figure 4:**
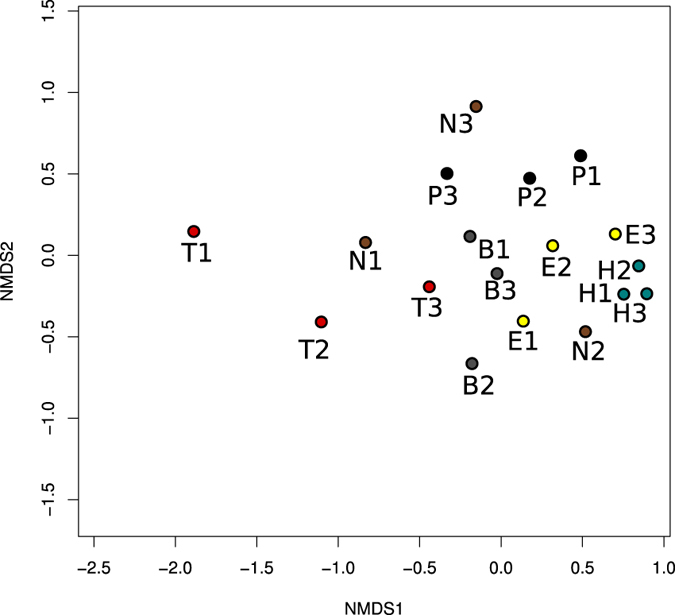
Non-metric multi-dimensional scaling (NMDS) plot showing similarities between gut bacterial communities from Colorado potato beetle larvae that were reared on different host plants (T, tomato; P, potato; E, eggplant; B, buffalobur; H, horsenettle; N, nightshade-fed larvae). Bray-Curtis dissimilarity matrix without singletons was used to generate NMDS coordinates (Stress: 0.137, R:^2^ 0.92).

To investigate the impact of host plant species on gut bacterial community structure of CPB larvae, we calculated Bray-Curtis dissimilarity distances and conducted an NMDS ordination. In the majority of cases, the bacterial communities associated with larvae fed on the same plant species were found in close proximity to one another on the NMDS plot, with the exception of those that fed on nightshade (Fig. 4). Bacterial communities of tomato-fed larvae and those of potato-fed larvae were closely clustered together within treatment but those larvae were separated from larvae fed on other host plants. Despite the clustering patterns observed on the NMDS plot, the bacterial communities associated with beetles fed on horsenettle, buffalobur, eggplant, nightshade, and potato did not differ significantly from the communities associated with insects fed on tomato (AMOVA, P > 0.05). Despite the lack of differences detected via AMOVA, 2D clustering analysis based on relative abundance of OTUs (with singleton removed) also confirmed the clustering patterns observed on the NMDS plot (Supplementary Fig. S5). When singletons were included in Bray-Curtis dissimilarity distances, overall clustering patters did not change (Supplementary Fig. S6). Only tomato-fed larvae showed high variation in bacterial communities. In these cases, two replicates were similar to one another, while the third replicate was more disparate, which suggests rare OTUs affect clustering pattern of bacterial communities.

**Figure 5:**
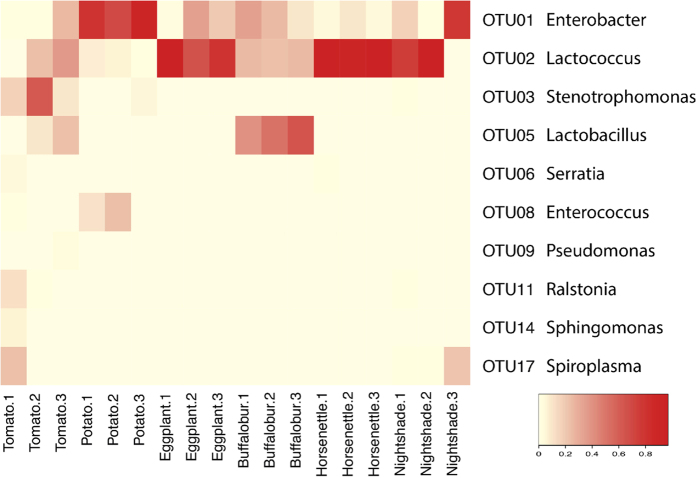
Heatmap showing relative abundance of the 10 most abundant OTUs from Colorado potato beetle larvae that were reared on different host plants.

The 10 most abundant OTUs among all samples comprised over 99% of the total sequences in all communities with the exception of those from tomato-fed larvae, which accounted for over 87% of the sequences (Fig. 5 and Supplementary Table S3). There were high variations in the relative abundances of these dominant OTUs among samples and among treatments. For example, the gut communities from tomato-fed larvae were dominated by one OTU from the genus *Stenotrophomonas* (OTU03) and one from the genus *Lactococcus* (OTU02), which comprised 29% and 20% of the reads, respectively. *Enterobacter* (OTU01) comprised 80% of the reads from potato-fed larvae. Eggplant-, horsenettle-, and nightshade-fed larvae were highly dominated by OTUs assigned to the genus *Lactococcus. Lactobacillus* (OTU05) was highly abundant in buffalobur-fed larvae. The relative abundances of bacterial taxa at the order level also showed high variation among samples and among treatments (Supplementary Fig. S7). These data suggest that the host plant can have impacts on several OTUs, particularly those that are present in the highest abundances.

### Differential abundance of defense-suppressing bacteria in larvae reared on *Solanum* hosts

**Figure 6:**
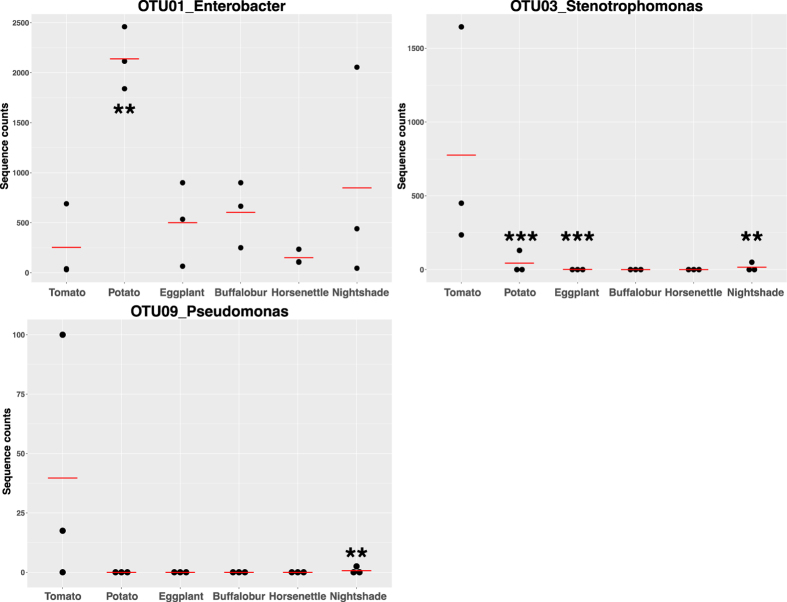
Sequence counts of three OTUs from Colorado potato beetle larvae that were reared on different host plants. Horizontal lines indicate the mean of three biological replicates. Asterisks indicate significant differences between tomato and other hosts. **P < 0.01; ***P < 0.001.

We previously identified three bacteria from tomato-fed larvae that suppress plant defenses^9^. To investigate how these defense-suppressing bacteria change in response to feeding on different host plants, we sequenced the V3-V4 regions of 16 S rRNA from colonies of these three bacteria and compared them to sequences in our 16 S amplicon library. Three OTUs showed > 99% sequence similarity to those of the defense-suppressing bacteria: *Enterobacter* (OTU01), *Stenotrophomonas* (OTU03), and *Pseudomonas* (OTU09). We then compared normalized sequence counts of these three OTUs in larvae fed on different host plants (Fig. 6). *Stenotrophomonas* (OTU03) and *Pseudomonas* (OTU09) were significantly more abundant in larvae reared on tomato compared to all other host plants (GLM, OTU03, tomato vs. potato, *z* = 32.05, *P* < 0.0001; tomato vs. eggplant, *z* = 8.03, *P* < 0.0001; tomato vs. nightshade, *z* = 2.81, *P* = 0.0049) (GLM, OTU09, tomato vs. nightshade, *z* = 3.11, *P* = 0.0019). *Enterobacter* (OTU01) was more abundant in potato-fed larvae than from other larvae (GLM, tomato vs. potato, *z* = −2.797, *P* = 0.0052). In larvae fed on most of the plant species except tomato, no reads from either *Stenotrophomonas* (OTU03) or *Pseudomonas* (OTU09) were detected and these samples were omitted from GLM testing. For example, no reads from either *Stenotrophomonas* (OTU03) or *Pseudomonas* (OTU09) were detected in all three of the gut communities from larvae reared on buffalobur and horsenettle. In addition, *Pseudomonas* (OTU09) was not detected in either potato- or eggplant-fed larvae.

### Bacterial communities differ between guts and oral secretion

Because it is likely that only a subset of the bacteria detected in the gut are secreted onto leaves and function as effectors to modify induced defenses, we compared the bacterial gut communities from CPB larvae fed on tomato with the communities in OS from tomato-fed larvae. Overall, there were considerable differences in the relative abundances of bacterial taxa at the order level among samples (Supplementary Fig. S8). The OS were highly dominated by *Enterobacter* (OTU01), *Acinetobacter* (OTU17), and *Lactococcus* (OTU02). *Enterobacter* (OTU01) in OS accounted for 42% of the total reads and was four times more abundant than it was in the CPB gut (Table 2). *Pseudomonas* (OTU09) in OS accounted for 3% of the total reads and was two times more abundant in OS compared to the gut. In contrast, OS contained a lower relative abundance of *Stenotrophomonas* (OTU03) compared to the gut.

## Discussion

Induced plant defenses are regulated by complex signaling pathways of plant hormones including JA and SA^1^. Antagonistic interactions between JA and SA are often involved in fine-tuning plant defenses and reducing the fitness costs associated with inducible defenses^2^. It is believed that this negative crosstalk is wide-spread in plants^7^. However, little is known about crosstalk between JA and SA in wild and cultivated *Solanum* plants. In this study, we found that MeJA application increased PPO activity in all *Solanum* plants compared to plants treated with EtOH or SA alone. Application of both JA and SA attenuated the induction of PPO activity. These data suggest that SA also has negative effects on the JA-signaling pathway in these *Solanum* plants, which is consistent with other studies. For example, JA application induced PPO activity, but application of JA and SA reduced PPO activity in tomato^10^. In addition, in a previous study, dual-application of JA and SA attenuated expression of *PPO1* in eggplant^12^.

Although MeJA induced PPO activity in all *Solanum* plants assayed, the magnitudes of PPO activity induced by MeJA and the level of attenuation of PPO activity by application of SA and MeJA were species-specific. In tomato, dual-application of MeJA and SA showed a lower level of PPO activity compared to plants treated with MeJA; however, the PPO activity levels in plants treated with both elicitors was still higher compared to control plants treated with EtOH. In contrast, PPO activity in other plants treated with both chemicals was similar to levels detected in control plants. This is likely because the magnitude of changes in PPO activity induced by MeJA in other plants were much smaller compared to tomato. Thus, it seems that other host plants analyzed in this study were less-responsive to MeJA elicitation compared to tomato.

We also detected the effect of antagonism between JA and SA on induced resistance to CPB larvae. In all *Solanum* plants, CPB larval growth on MeJA-treated plants was significantly lower compared to plants treated with EtOH or SA alone. This finding is consistent with a previous report, which showed that CPB growth was compromised on MeJA-treated potato^29^. Further, larval growth was also attenuated on tomato and horsenettle treated with both SA and MeJA, suggesting that JA-induced resistance was suppressed by SA signaling. Additionally, larval growth was well correlated with PPO activity profiles, suggesting that PPO is likely involved in plant response to CPB in most plant species included in this study except horsenettle. The manipulation of defensive proteinase inhibitor proteins or secondary metabolites by MeJA and SA could explain larval growth pattern on horsenettle. Other studies identified negative interactions between JA and benzothiadiazole (BTH, a functional analog of salicylic acid) on herbivore performance. For example, the relative growth rates of *Spodoptera exigua* and *Trichoplusia ni* caterpillars fed on tomatoes treated with both JA and BTH were lower than those observed in caterpillars reared on control plants, but was higher than the relative growth rate of insects reared on tomatoes treated with JA alone^10^,^30^. Taken together, these data indicate that JA-SA antagonism is present in *Solanum* plants, and thus may be exploited by herbivores and their symbiotic bacteria^9^,^31^,^32^.

Diets have, in some cases, been shown to induce changes in microbial communities associated with insects and these changes in bacterial communities could play an important role in mediating interactions between plants and insects^33^. We previously showed that three symbiotic bacteria are responsible for suppression of JA-mediated plant defenses^9^. This manipulation of plant defenses was attributed to negative crosstalk between JA and SA-signaling pathways. The current study demonstrates that host plants influence the gut microbial community of CPB larvae associated with suppression of plant defenses. For example, when tomato-fed larvae were fed on different host plants, AB-untreated larvae suppressed plant defenses in comparison to AB-treated larvae. Interestingly, this response was host plant specific. Only tomato- and potato-fed larvae suppressed plant defenses in tomato and potato, respectively. This phenomenon was not observed in the four other plant species tested in this study. Because we showed that there are antagonistic interactions between JA and SA in all *Solanum* host plants we tested, it is unlikely that lack of suppression of plant defenses in other plants is due to the absence of defense signaling pathways in these plants. These data suggest that host mediated differences in larval CPB gut microbiota may be linked to the insects’ ability to manipulate host plant defenses.

In order to manipulate plant defenses, a certain amount and species of defense-suppressing bacteria must be delivered to leaves through deposition of OS. The amount of OS deposited onto a leaf and the abundance of defense-suppressing bacteria in these secretions varied tremendously depending on which host plant the insects fed. Thus, variations in abundance and concentration of these defense-suppressing bacteria in OS could determine whether or not the insect is able to successfully suppress JA-mediated defenses. In this study, tomato-fed larvae had higher relative abundances of bacteria from the defense-suppressing taxa *Stenotrophomonas* (OTU03) and *Pseudomonas* (OTU09) compared to all other taxa. Potato-fed larvae harbored a greater abundance of *Enterobacter* (OTU01) than larvae reared on other host plants. qPCR measurement of the *rpoD* gene confirmed that *Pseudomonas* (OTU09) was secreted onto potato by potato fed larvae. Interestingly, eggplant-, buffalobur-, horsenettle-, and nightshade-fed larvae did not decrease PPO activity in their corresponding plants but those larvae had similar abundance of *Enterobacter* (OTU01) to tomato-fed larvae which decreased PPO activity. Because suppression of PPO activity in tomato by bacteria in OS is dose-dependent^9^, it is likely that abundance of *Enterobacter* (OTU01) in those larvae is not enough to modify plant signaling pathways. These data suggest that *Stenotrophomonas* (OTU03) and *Pseudomonas* (OTU09) in tomato-fed larvae and *Enterobacter* (OTU01) and *Pseudomonas* (OTU09) in potato-fed larvae were primarily responsible for suppression of plant defenses in these two hosts.

16 S rRNA amplicon sequencing is a rapid and cost effective way to characterize complex microbial communities, but there are several limitations regarding this method. Sequencing artifacts and errors as well as primer biases can introduce noise into the analysis and prevent accurate estimation of OTUs^34^. For example, some of the dominant bacterial taxa in the *Pyrrhocoris apterus* gut were not detected by 454 pyrosequencing, but high relative abundances of these bacteria were detected by qPCR^35^. More importantly, some bacterial taxa with low abundance may not be detected at all^36^. For example, in our study reads derived from *Pseudomonas* (OTU09) were not identified from the guts of potato-fed CPB larvae; however qPCR using the *rpoD* gene demonstrated the presence of *Pseudomonas* (OTU09) on potato leaves secreted by potato-fed larvae. This discrepancy may be due to low relative abundance of *Pseudomonas* (OTU09) and more highly abundant *Enterobacter* (OTU01) in gut samples compared to OS samples. It is also possible that differences in relative abundance between OS and gut samples may explain this discrepancy. For example, the abundance of *Pseudomonas* (OTU09) in OS was approximately twice as high than was detected in gut samples.

Microbial composition and structure in guts vary when caterpillars feed on different host plants^27^,^37^,^38^,^39^. Some of the variation may be due to plant secondary metabolites, which were shown to influence microbial diversity and relative abundances of several major taxa in the gut of woodrats^40^. Glycoalkaloids (GAs) are secondary metabolites prevalent in the Solanaceae and are involved in defense against herbivores and plant pathogens^41^,^42^,^43^. Each *Solanum* plant has a different profile of major GAs, including α-tomatine in tomato, α-chaconine and α-solanine in potato, α-solamargine and α-solasonine in eggplant, and α-solasonine and α-solamargine in buffalobur. Because GAs may possess variable antibacterial activities, they could play a role in shaping microbial community and the relative abundance of several OTUs in gut samples. The structure of CPB gut communities was influenced by the host plant, although this effect was not statistically significant due to the high variation in the bacterial communities in tomato-fed larvae. Some bacteria may be highly affected by plant secondary metabolites. For example, phenolic glycosides and condensed tannin in leaves of aspen trees (*Populus tremuloides*) differentially affected relative abundance of two dominant bacteria in the midguts of *Lymantria dispar* caterpillars^44^. The relative abundance of *Ralstonia* sp. increased but relative abundance of *Acinetobacter* sp. decreased when caterpillars fed on leaves containing high levels of condensed tannins. In contrast, the relative abundance of *Ralstonia* sp. was negatively correlated with the concentration of phenolic glycosides. GAs could also affect gut physiology and, in turn, differentially affect the ability of bacteria to colonize or persist within the gut.

In addition to secondary metabolites, physical properties of different host plants may influence bacterial communities. Leaf toughness and trichomes could modify the nutritional quality of plants, which could directly impact larval growth and physio-chemical interactions between bacteria and the insect host. For example, stellate trichomes in *S. sysymbriifolium* could puncture the peritrophic membrane in the gut of beetle larvae (*Gratiana spadices*)^45^. Damage to the peritrophic matrix negatively affected digestion and absorption of nutrients and consequently disturbed the growth and development of the caterpillar^46^,^47^. In our current study, when CPB larvae were reared on buffalobur and horsenettle, which both have stellate trichomes, the relative abundances of members of Lactobacillales, *Lactococcus* (OTU02) and *Lactobacillus* (OTU05) increased compared with larvae reared on tomato. *Lactobacillus plantarum* in the gut of *Drosophila* was associated with larval growth on a nutrient poor diet^48^. *Lactococcus lactic* in the gut of red palm weevil (*Rhynchophorus ferrugineus*) is involved in digestion of polysaccharides and sucrose^49^. Thus, the increase in *Lactococcus* (OTU02) and *Lactobacillus* (OTU05) observed in our study may allow the insect to compensate for reduced nutrient absorption caused by trichomes. It would be important to investigate which properties in plants drive changes in specific gut microbiota.

We also demonstrated that the relative abundances of major bacterial OTUs changed when larvae adapted to feed on tomato were fed on other host plants. However, the bacterial communities from larvae in a natural population likely differ from the community we observed in our lab population used for this study. Substantial differences in insect bacterial communities between lab and field populations have been reported^27^,^50^. The insect genotype can also shape the gut microbial composition^51^. Thus, it is likely that the gut microbiota from potato-fed larvae in our lab colony differs from that of larvae reared in a potato field.

We cannot exclude the possibility that the intensity of induced defenses plays an important role in shaping gut microbiota in natural populations. In our study, larvae were fed on detached leaves instead of whole plants and, therefore, how the strength of induced defenses impacts on larval gut microbiota was not quantified in this study. Further, the extent to which defenses are induced by herbivory can differ depending on plant species and environmental conditions such as light intensity, temperature, and nutrient availability^52^.

CPB appears to have expanded its host range from buffalobur to cultivated *Solanum* plants as they migrated from its native range in central Mexico to North America^53^. Herbivore-associated bacteria could have played a role in adaptation to new hosts and host range expansion^20^,^33^,^54^. It is possible that the association of defense-suppressing bacteria with CPBs helped them adapt to a diverse range of *Solanum* host plants in wild and agricultural systems. Thus, it is noteworthy to investigate the underlying mechanism of acquisition and maintenance of the gut bacteria and how gut microbiota, including plant defense-suppressing bacteria, differ among geographic regions.

To our knowledge this is the first study to investigate the effect of negative crosstalk between JA and SA on induced defenses and resistance in wild *Solanum* host plants to CPB. We demonstrated that antagonistic interactions between JA and SA occurs in wild hosts of CPB and that bacteria associated with CPB larvae are also capable of suppressing defenses in these wild hosts. More importantly, differences in relative abundances of defense-suppressing bacteria, as well as differences in gut community composition triggered by feeding on different host plants, can strongly impact the ability of CPB to overcome plant defenses. Due to the importance of the bacterial community associated with insect guts and OS in plant defense suppression, further research is warranted to determine how symbiotic bacteria are transmitted and to decipher the underlying mechanisms by which CPB can successfully manipulate plant defenses for their own benefit and adapt to new host plants under natural conditions.

## Materials and Methods

### Plants and Insects

Seeds of tomato (*Solanum lycopersicum* cv. Betterboy) and eggplant (*S. melongena* cv. Black Beauty), nightshade (*S. dulcamara*) were purchased from commercial suppliers (Harris Seeds, Ferry Morse, and Horizon Herbs, respectively). Seedlings of horsenettle (*S. caroliense*) germinated from field-collected seeds were kindly provided by Rupesh Kariyat. Potato tubers (*S. tuberosum* cv. Atlantic) were kindly provided by Michael Peck. Seeds of buffalobur (*S. rostratum*, PI420997) were obtained from the US Department of Agriculture-Agricultural Research Service National Genetic Resources Program. Seeds were planted in Pro-mix potting soil (Premier Horticulture) in a greenhouse with a photoperiod of 16 hL:8 hD. Seedlings with at least one true leaf were transplanted into 4-in pots and fertilized with 3 g of Osmocote plus (15-9-12, Scotts). After one month of growth, plants were used for herbivore and chemical elicitor treatments. The colony of CPB was maintained as described previously^9^. Briefly, eggs were hatched and larvae were reared on detached tomato leaves (cv. Better Boy) in a growth chamber under conditions of 16 L:8D and 27 °C. Adults were reared on tomato plants in a mesh cage (W × L × H = 75 × 63 × 88 cm) in a greenhouse.

To test the effects of host plants on plant defenses and on bacterial community structure and composition, eggs from the lab colony that were maintained on tomato were randomly selected and placed on the six different host plants. The larval colonies for each host plant were maintained separately in a growth chamber. Fourth instar larvae that had been reared on detached leaves for 7–8 days were collected for DNA–extraction and stored at −80 °C until used.

### Chemical elicitor treatment

To determine whether negative crosstalk between JA and SA occurs in CPB host plants, methyl jasmonate (MeJA) and salicylic acid (SA) were applied to tomato, potato, eggplant, buffalobur, horsenettle, and nightshade. Both 0.1 mM MeJA (Bedoukian Research) and 1 mM SA (Sigma) were dissolved in 0.8% ethanol (EtOH). The concentrations of both elicitors were selected based on a previous experiment where strong negative crosstalk between MeJA and SA was detected in tomato (data not shown). The elicitors were sprayed on plants until runoff. Control plants were sprayed with 0.8% EtOH. To measure polyphenol oxidase (PPO) activity, leaf tissue (100 mg) from the terminal leaflets of the third leaves were harvested 48 h after treatment, frozen in liquid nitrogen and stored at -80 °C until used. For potato, the sixth leaf from the bottom was used.

### Herbivore treatment

To reduce bacterial titers in CPB larvae, we used an antibiotic (AB) solution as described previously^9^. Briefly, AB solutions were prepared in 50 mL of MilliQ water and contained the following anti-bacterial agents: 0.01 g neomycin sulfate (MPbio), 0.05 g aureomycin (Bioserv), and 0.003 g streptomycin (Sigma). Each third instar larva was fed on three leaves treated with the AB solution or MilliQ water (control) for a 3-day period.

To investigate if symbiotic bacteria suppress plant defenses, one AB-treated or untreated larva was placed on the terminal leaflet of the third leaf from the bottom using a clip cage for each plant. For potato, the sixth leaf from the bottom was used. Undamaged plants received an empty cage. Once each larva consumed 100% of the confined area, the larva and cage were removed. Leaf tissue (100 mg) from the damaged leaflets was harvested 48 h after insect infestation, frozen in liquid nitrogen and stored at −80 °C until use.

### Polyphenol oxidase (PPO) activity

PPO is a well-known JA-inducible protein and has a negative effect on CPB growth^55^. PPO activity was selected to measure JA-induced plant defense in *Solanum* hosts. PPO activities were measured 48 h after treatment using caffeic acid (Sigma) as the substrate as described previously^56^. Total protein was measured using the Bradford assay^57^ with bovine serum albumin (Sigma) as a standard.

### Bioassay

To investigate the effect of chemical elicitors on CPB performance, the growth rates of neonate larvae were measured. We excised the third and fourth leaves from each plant that was sprayed with either SA- or JA- or both elicitors simultaneously. For potato, the sixth and seventh leaves from the bottom were used. Individual neonates were placed on detached leaves (ca. 0.3–0.5 mg) in a diet cup containing 1% agar and were allowed to feed for 5 days.

### OS collection and quantification of OS deposited onto leaves

OS were collected from approximately 300 fourth instar larvae reared on tomato leaves. Crude OS were stored at −80 °C until used. The amount of OS secreted onto each leaf was quantified using a fluorescent dye as described previously^58^. Briefly, fourth instar larvae that were reared on one of six different host plants were fed on the corresponding leaves containing the dye overnight. Damaged sections were examined 10 min after the larvae fed on new leaves.

### DNA extraction, quantitative real time polymerase chain reaction (qPCR) and 16 S rRNA amplicon sequencing

To quantify defense-suppressing bacteria secreted onto potato leaves by larvae that were reared on tomato and potato, we measured abundance of *rpoD* (sigma factor subunit of RNA polymerase) gene of *Pseudomonas* sp. using gene specific primers as described previously^9^. Briefly, total DNA was extracted from leaves that were damaged by AB-treated or untreated larvae using the DNeasy Plant Mini kit (Qiagen) following the manufacturer’s protocol. Levels of *rpoD* abundance were measured using 100 ng of DNA and the *rpoD* primer pair rpoDF (5′-GGTCGTGCCCACAAGGAA-3′)/rpoDR (5′-AACTGCTTGGGTACCAGCTTGA-3′). A standard curve was generated using a serial dilution of plasmids containing one copy of the target sequence. Absolute quantification of *rpoD* copy number was calculated using threshold values (Ct) taking total DNA concentration into account^59^.

To characterize the gut microbial communities associated with insects feeding on different hosts, larvae were first surface-sterilized with 10% bleach, 70% ethanol, and three washes of autoclaved water. After surface sterilization, larvae were dissected under the dissecting microscope with sterile tools to remove gut tissues. Whole guts from three to four larvae were pooled together for DNA isolation and three replicate pools were collected for insects feeding on each plant species. Gut tissues were homogenized in liquid N_2_ and total DNA was extracted using the FastDNA Spin kit for Soil DNA Extraction kit (MP Biomedicals) following the manufacturer’s protocol. To extract DNA from OS samples, approximately 1 mL of OS was centrifuged at 11,000 *g* for 10 min at room temperature and the pellets were used for DNA isolation as described above. We amplified the V3-V4 region of the 16 S rRNA gene using the primer pair of 347 F (5′-GGAGGCAGCAGTRRGGAAT-3′)/803 R (5′-CTACCRGGGTATCTAATCC-′3), which contained Illumina TruSeq DNA adapters and barcodes. PCR was performed in a 25 μL reaction volume containing 2.5 μL of DNA (40–50 ng), 5.0 μL of each primer (1 μM), and 12.5 μL of 2x KAPA HiFi HotStart ReadyMix (Kapa Biosystems). The PCR conditions were as follows: 95 °C for 5 min, followed by 30 cycles of 95 °C for 1 min, 53 °C for 1 min, and 72 °C for 90 sec, and 72 °C final extension for 7 min. The quality of the PCR products was verified by gel electrophoresis. Negative controls for DNA extraction were conducted using sterile water; no amplified PCR products were detected. The amplicons from all samples were pooled together and were sequenced on the Illumina MiSeq instrument at Penn State Genomics Core Facility (University Park, PA) to a depth of approximately 500,000 300 × 300 bp reads per sample.

Amplicons were processed using the program mothur (version 1.32.0). Overlapping paired end reads were merged using the ‘make.contigs’ command. Contigs between 430 and 460 bp in length were retained if they overlapped by a minimum of 75 bp, zero mismatches were detected in the overlapping region, and no more than one ambiguous base was detected throughout the entire consensus sequence. Sequences were de-replicated using the ‘unique.seqs’ command and then aligned to the Silva reference alignment (release 123) using the Needleman aligner with the flip = T option. The alignment was trimmed to 430 bp using the ‘screen.seqs’ command. Reads that did not align to the V3-V4 region of the Silva reference alignment were discarded and reads that potentially contained sequencing errors, defined as sequences that had a ≤ 2 base-pair mismatch with at least one more highly abundant sequence, were grouped together using the ‘pre.cluster’ command with the diffs = 2 option. Chimeras were removed using the ‘chimera.uchime’ command and the self = T option. In this manner, the more highly abundant reads were used as templates to screen for chimeras. Operational taxonomic units (OTUs) were predicted using the average neighbor algorithm and a genetic Jukes-Cantor corrected genetic distance of 0.03. OTUs were taxonomically classified using RDP Classifier and an 80% confidence threshold. OTUs classified as chloroplast, mitochondrial, unknown, Archaeal, or Eukaryotic in origin were discarded. To remove any additional plant or insect derived reads that may not have been adequately classified by RDP, the consensus sequence for each OTU was compared to the non-redundant nucleotide database using blastn + with an e-value threshold of 1E-10, retaining the top 10 highest scoring blast matches. OTUs whose top 10 blast matches were exclusively non-bacterial in origin were also eliminated from the analysis. Prior to running any comparative analysis, the same number of reads (n = 2647) was randomly subsampled from each community to prevent differences in library yields from driving similarities and differences in various ecological indices. Raw MiSeq paired end reads are deposited under NCBI’s Sequence Read Archive (SRA) SRR3723123 to SRR3723141 under BioProject PRJNA326955.

To determine which OTUs corresponded to the three defense-suppressing bacteria detected previously^9^, the V3-V4 region of 16 S rRNA gene was amplified with DNA collected from bacterial isolates from the defense-suppressing bacterial isolates as described previously^9^. The PCR products were sequenced bidirectionally and compared to reads in MiSeq library using blastn+ . In all cases, a single OTU with ≥ 99% nucleotide similarity to the V3-V4 region of the defense-suppressing bacteria were readily identified, indicating a high likelihood that these OTU are definitively derived from these defense-suppressing bacteria and were unlikely derived from a close strain of the bacteria.

### Statistical and bacterial community analyses

PPO activity, larval mass, and *rpoD* copy numbers were analyzed using one-way ANOVA followed by Fisher’s Least Significant Difference (LSD) test or an unpaired t-test. Larval mass and *rpoD* copy numbers were log-transformed to meet the assumptions of ANOVA and t-test. For alpha and beta diversity index measurements, all sequences with and without singletons were randomly subsampled (without replacement) to the same depth (n = 2647) with 1,000 iterations using the ‘sub.sample’ command in mothur. Good’s coverage, Shannon and Simpson diversity indexes, and Chao1 richness were calculated using the ‘summary.single’ command in mothur. To determine whether feeding in different hosts causes major changes in community structure, a Bray-Curtis dissimilarity matrix was calculated and analysis of molecular variance (AMOVA) was used. An NMDS ordination was used to cluster the samples by similarity using the ‘nmds’ command in mothur. Rarefaction curves were computed using the ‘rarefaction.single’ command in mothur. Non-parametric Wilcoxon rank tests were used to determine whether community richness or diversity indexes were different and were performed in R v.3.2.1^60^. A heatmap was generated in R using the relative abundance of the top 10 OTUs. To investigate whether the abundance of defense-suppressing bacteria differs in response to diet, a generalized linear model with negative binomial errors was employed in R. All the statistical tests on bacterial community analyses were conducted to access differences between data from tomato fed larvae and those from larvae fed on other host plants.

## Additional Information

**How to cite this article**: Chung, S. H. *et al*. Host plant species determines symbiotic bacterial community mediating suppression of plant defenses. *Sci. Rep.* **7**, 39690; doi: 10.1038/srep39690 (2017).

**Publisher's note:** Springer Nature remains neutral with regard to jurisdictional claims in published maps and institutional affiliations.

## Supplementary Material

Supplementary Information

## Figures and Tables

**Table 1:** Alpha diversity indexes without singletons from Colorado potato beetle larvae that were reared on different host plants.

Host	Coverage	OTUs	Chao1	Shannon	1/Simpson
Tomato-1	0.999	67	67.2	2.87	9.34
Tomato-2	1.000	80	83.8	1.18	2.30
Tomato-3	1.000	60	62.8	1.51	3.79
(mean ± SEM)		(69.0 ± 5.9)	(71.3 ± 6.4)	(1.85 ± 0.52)	(5.16 ± 2.14)
Potato-1	1.000	47	68.9	0.67	1.50
Potato-2	1.000	42	52.9	0.82	1.85
Potato-3	1.000	33	40.5	0.32	1.15
(mean ± SEM)		(40.7 ± 4.1)	(54.1 ± 8.2)	(0.61 ± 0.15)	(1.50 ± 0.20)
Eggplant-1	0.999	46	49.0	0.17	1.06
Eggplant-2	1.000	27	31.0	0.66	1.80
Eggplant-3	1.000	19	21.5	0.55	1.53
(mean ± SEM)		(30.7 ± 8.0)	(33.8 ± 8.1)	(0.46 ± 0.15)	(1.46 ± 0.22)
Buffalobur-1	0.999	46	65.4	1.16	2.99
Buffalobur-2	1.000	41	52.0	1.08	2.64
Buffalobur-3	1.000	43	53.5	0.94	2.08
(mean ± SEM)		(43.3 ± 1.4)	(57.0 ± 4.2)	(1.06 ± 0.06)	(2.57 ± 0.26)
Horsenettle-1	1.000	15	17.0	0.26	1.13
Horsenettle-2	1.000	17	26.0	0.30	1.19
Horsenettle-3	1.000	15	22.0	0.20	1.10
(mean ± SEM)		(15.7 ± 0.7)	(21.7 ± 2.6)	(0.25 ± 0.03)	(1.14 ± 0.03)
Nightshade-1	1.000	47	50.5	0.93	1.70
Nightshade-2	1.000	27	30.5	0.16	1.06
Nightshade-3	0.999	45	65.0	0.58	1.54
(mean ± SEM)		(39.7 ± 6.4)	(48.7 ± 10.0)	(0.56 ± 0.22)	(1.44 ± 0.19)

**Table 2:** Relative abundance (%) of the 10 most abundant OTUs in the gut and OS from Colorado potato beetle larvae that were reared on tomato.

		Gut-1	Gut-2	Gut-3	Gut*	OS	Ratio of OS to Gut
Enterobacter	OTU01	1.51	1.13	26.03	9.56	42.09	4.4
Lactococcus	OTU02	0.11	23.54	36.83	20.16	10.54	0.5
Stenotrophomonas	OTU03	16.92	62.18	8.84	29.32	8.84	0.3
Enterobacteriaceae	OTU04	0.38	0.08	0.49	0.31	1.47	4.7
Lactobacillus	OTU05	—	8.99	23.16	10.72	—	—
Serratia	OTU06	4.00	—	0.19	1.40	2.30	1.6
Enterococcus	OTU08	1.66	—	—	0.55	0.53	1.0
Pseudomonas	OTU09	0.68	0.04	3.78	1.50	3.14	2.1
Ralstonia	OTU11	12.32	1.17	0.30	4.60	—	—
Sphingobacterium	OTU12	—	—	—	—	9.07	—
Sphingomonas	OTU14	6.46	0.04	0.04	2.18	—	—
Acinetobacter	OTU15	—	—	0.08	0.03	17.87	709.5
Spiroplasma	OTU17	22.48	—	—	7.49	0.19	0.0

^*^Average of relative abundance of three biological replicates.
