# Supplementary material for: Host plant species determines symbiotic bacterial community mediating suppression of plant defenses
Source: Sci Rep. 2017 Jan 3;7:39690. doi: 10.1038/srep39690 (PMC5206732; doi:10.1038/srep39690)
Supplement: Supplementary Information [file srep39690-s1.pdf]

Host plant species determines symbiotic bacterial community mediating suppression of plant defenses

Seung Ho Chung[1], Erin D. Scully[2], Michelle Peiffer[3], Scott M. Geib[4], Cristina Rosa[5], Kelli Hoover[3], Gary W. Felton[3]

[1]Department of Entomology, Cornell University, Ithaca, NY 14853, USA
[2]Stored Product Insect and Engineering Research Unit, USDA-Agricultural Research Service, Center for Grain and Animal Health Research, Manhattan, KS 66502, USA
[3]Department of Entomology, Pennsylvania State University, University Park, PA 16802, USA
[4]Tropical Crop and Commodity Protection Research Unit, USDA-ARS Daniel K. Inouye Pacific Basin Agricultural Research Center, Hilo, HI 96720, USA
[5]Department of Plant Pathology and Environmental Microbiology, Pennsylvania State University, University Park, PA 16802, USA

Correspondence and requests for materials should be addressed to G.W.F. (email: gwf10@psu.edu)

Supplementary Information:

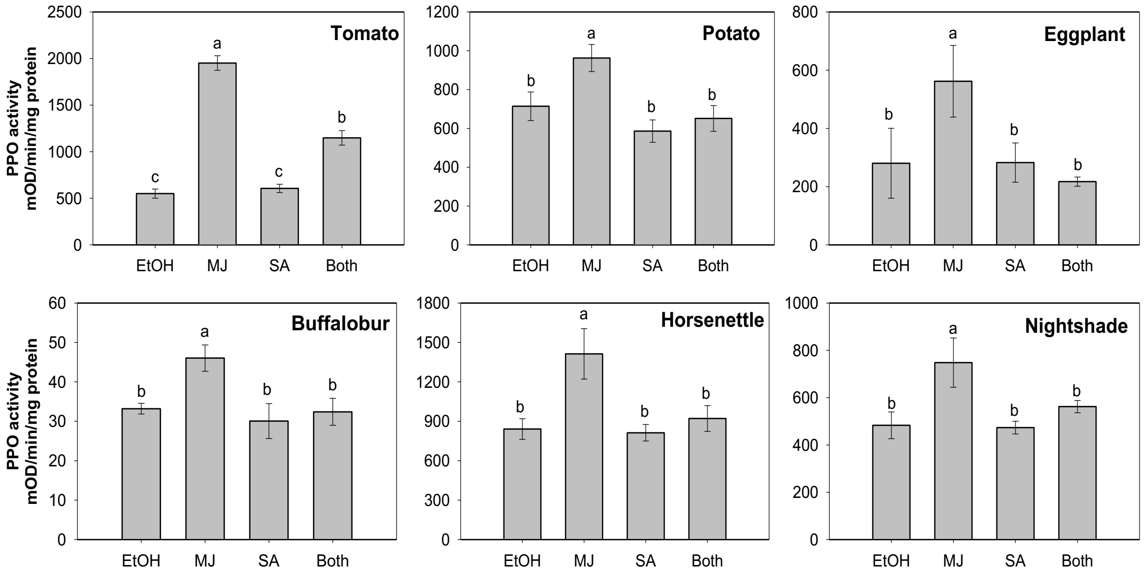

Fig. S1. Polyphenol oxidase (PPO) activity in plants sprayed with methyl jasmonate (MJ), salicylic acid (SA), both elicitors (Both), or the ethanol (control). PPO activity was measured 48 h after treatment. Values are means ± SEM. Different letters above the bars represent significant differences (ANOVA, $P < 0.05$, $N = 5\text{-}10$).

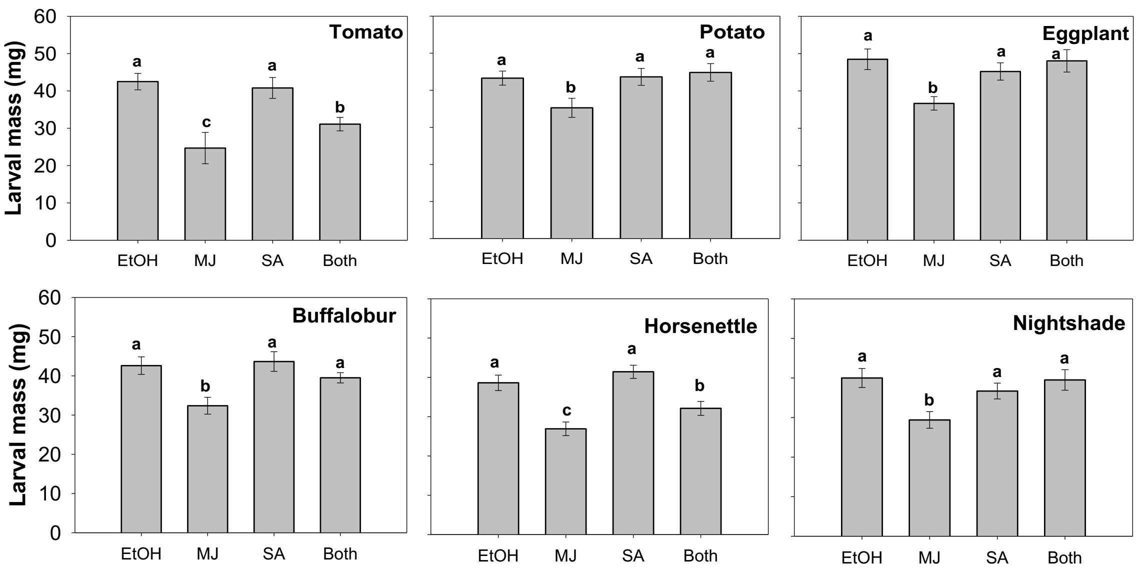

Fig. S2. Weight of larvae fed on plants sprayed with methyl jasmonate (MJ), salicylic acid (SA), both elicitors (Both), or the ethanol (control). Neonates were allowed to feed on excised leaflets for 5 days and larval mass was determined. Values are means ± SEM. Different letters above the bars represent significant differences (ANOVA, $P < 0.05$, $N = 20\text{-}30$).

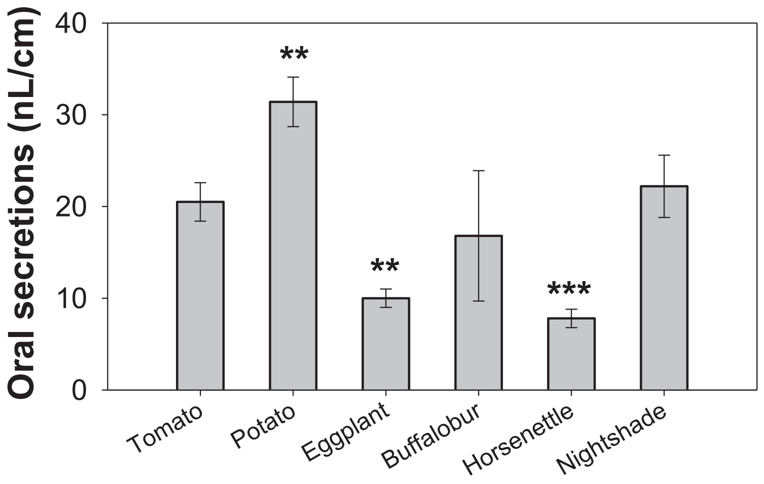

Fig. S3. The amount of oral secretions (OS) secreted by Colorado potato beetle larvae that were reared on different host plants. Values are means ± SEM (N = 5-10). Asterisks indicate significant differences compared with tomato. ** $P < 0.01$; *** $P < 0.001$.

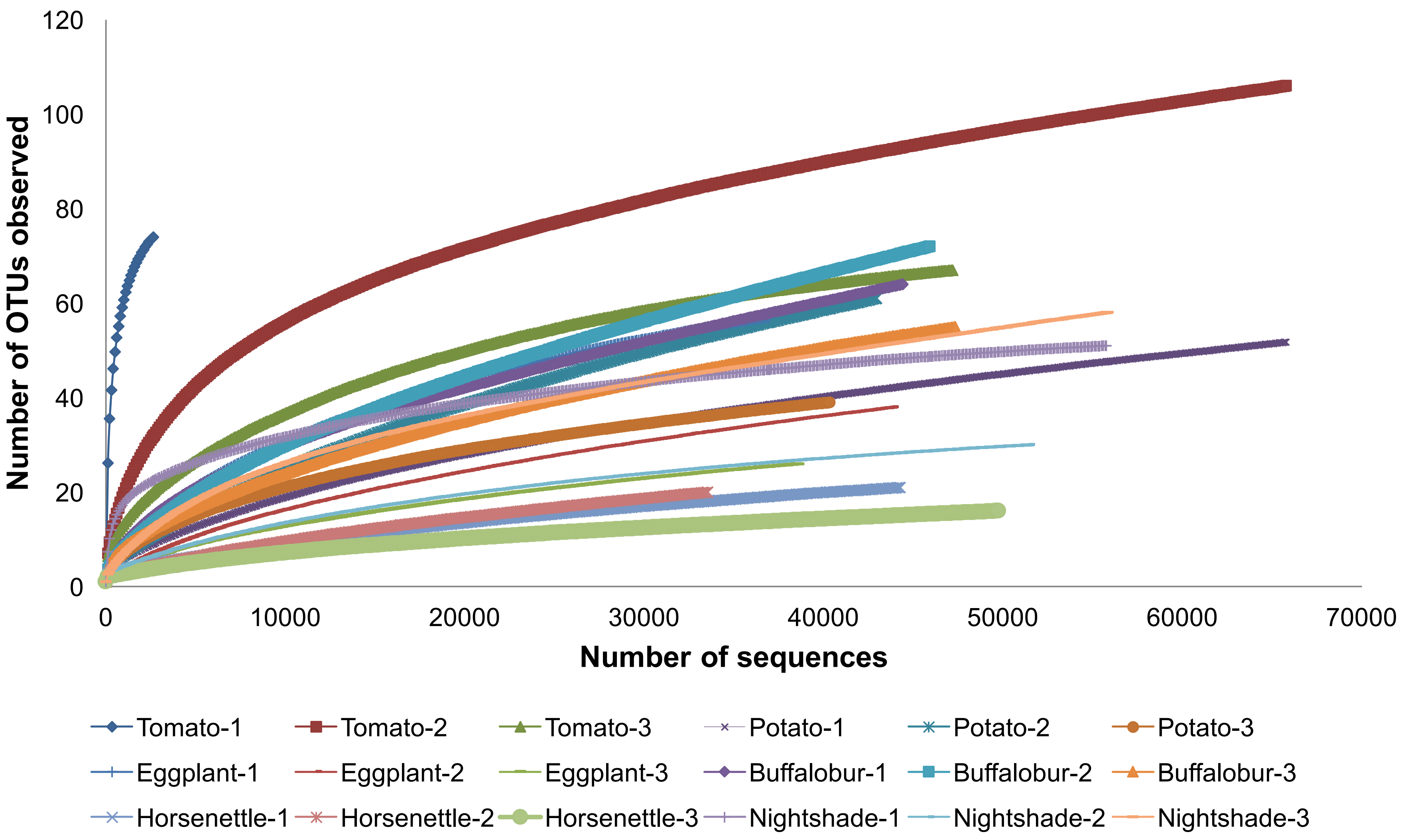

Fig. S4. Rarefaction curves for 16S gut bacterial communities of Colorado potato beetle larvae that were reared on different host plants.

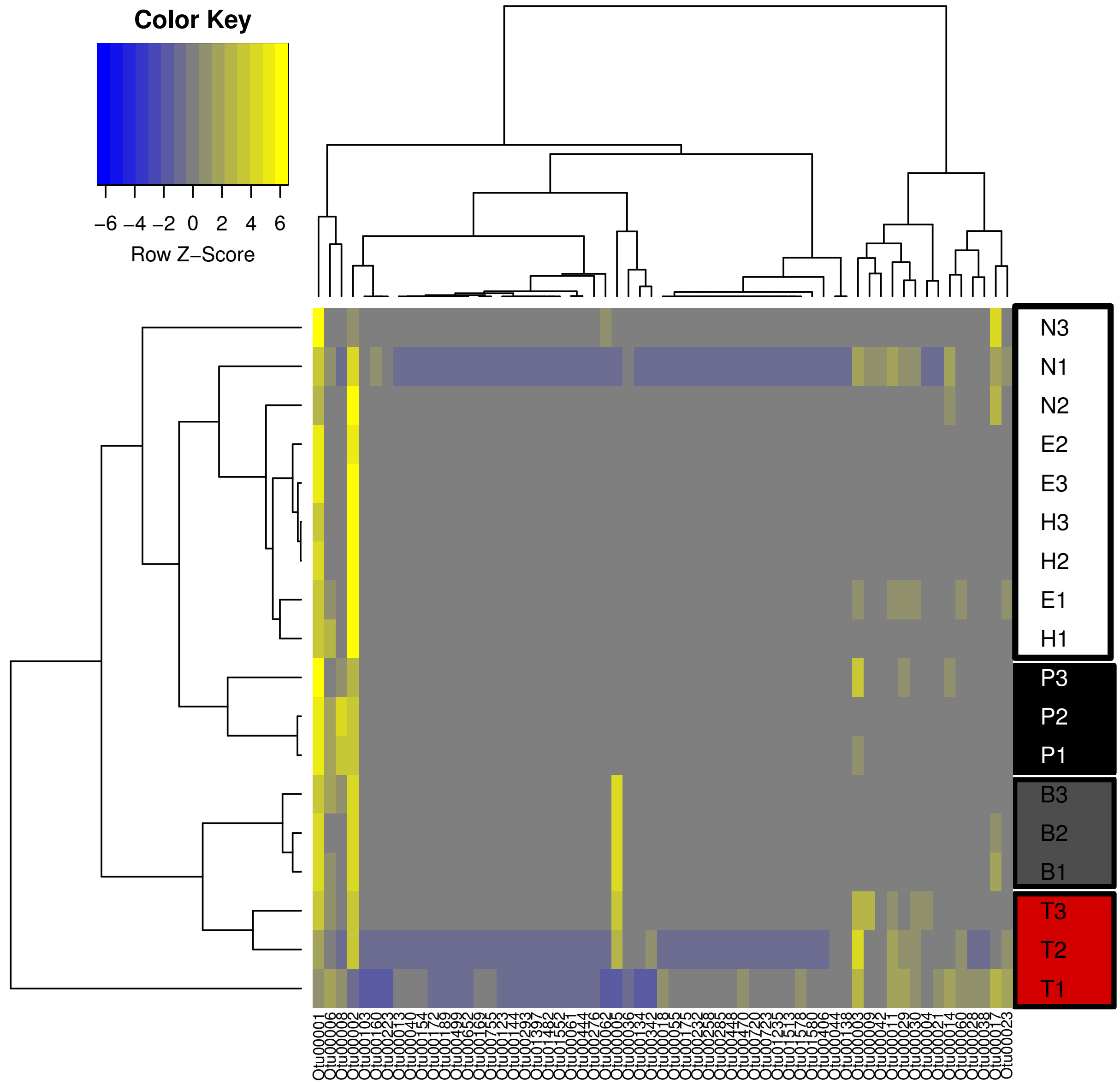

Fig. S5. 2D hierachial clustering dendrogram and heatmap showing realtive abudance of OTUs over 1% of total reads from Colorado potato beetle larvae that were reared on different host plants (T, tomato; P, potato; E, eggplant; B, buffalobur; H, horsenettle; N, nightshade-fed larvae). OTU table was presented in Table S4.

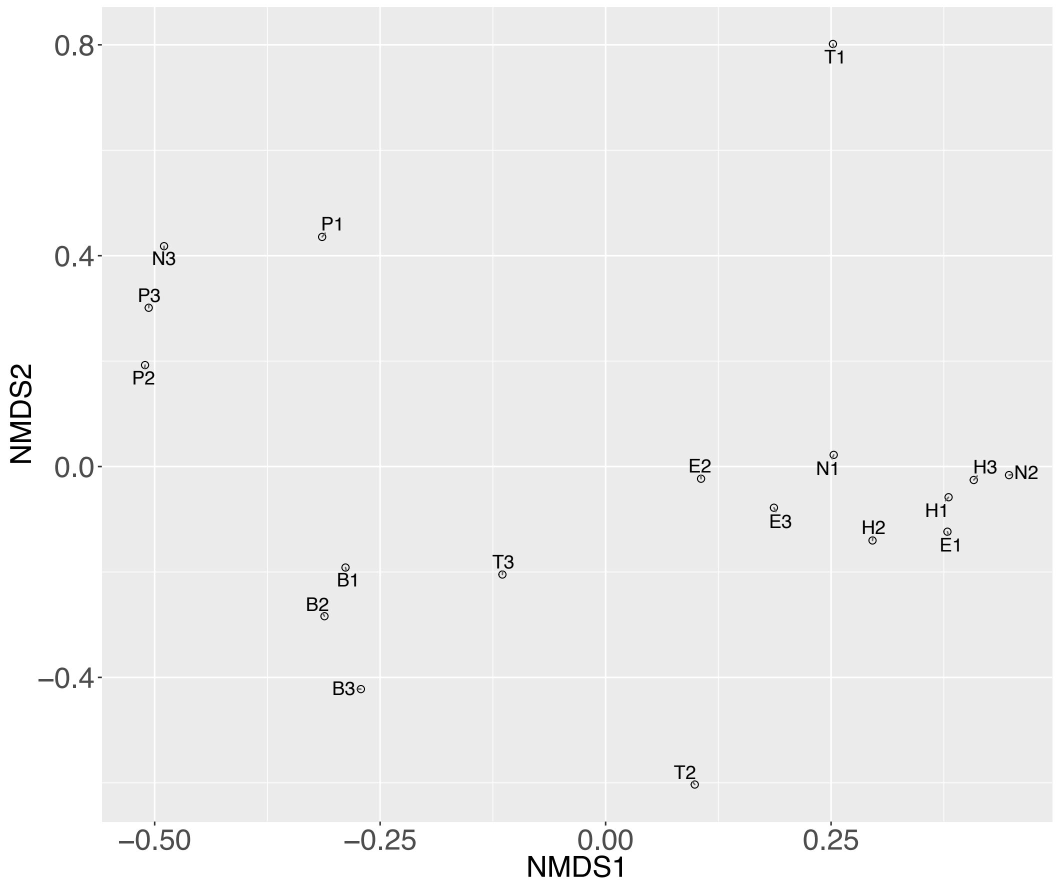

Fig. S6. Non-metric multi-dimensional scaling (NMDS) plot showing similarities between gut bacterial communities from Colorado potato beetle larvae that were reared on different host plants (T, tomato; P, potato; E, eggplant; B, buffalobur; H, horsenettle; N, nightshade-fed larvae). Bray-Curtis dissimilarity matrix with singletons was used to generate NMDS coordinates (Stress: 0.141, R^2: 0.92).

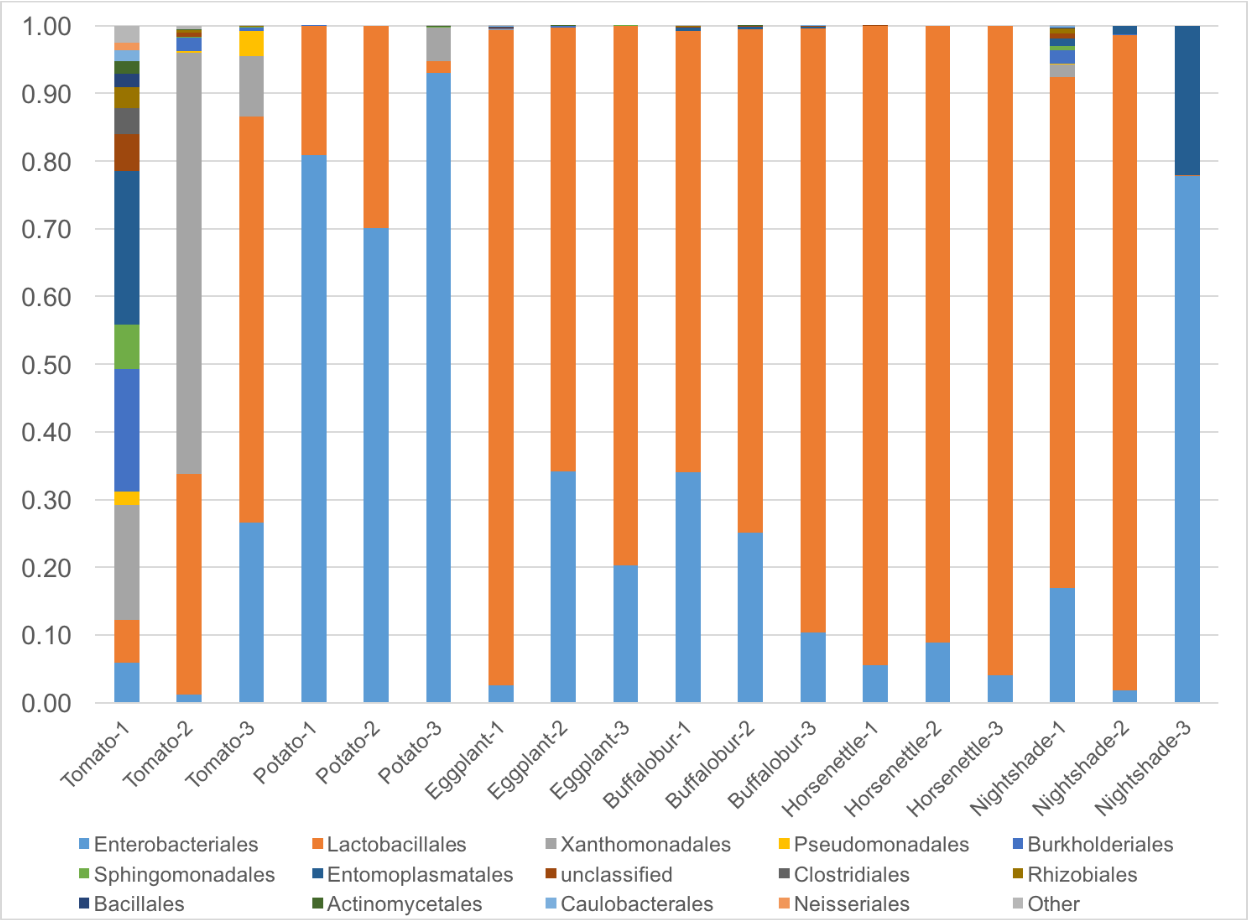

Figure S7. Relative abundance of bacterial communities at the order level from Colorado potato beetle larvae that were reared on different host plants. Taxa with relative abundance less than 0.01 were assigned to other.

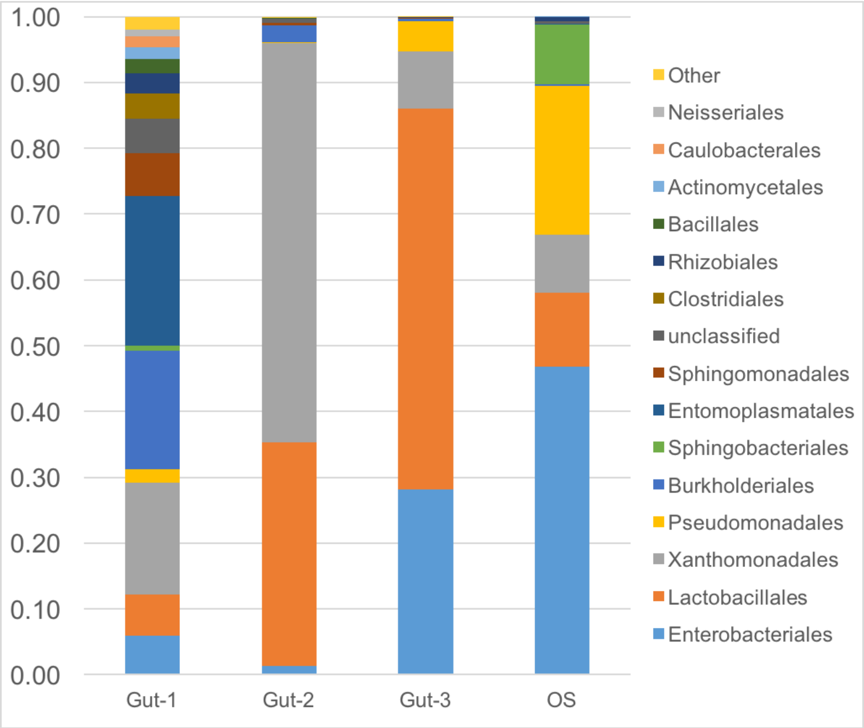

Figure S8. Relative abundance of bacterial communities at the order level in guts and OS from Colorado potato beetle larvae that were reared on tomato. Taxa with relative abundance less than 0.01 were assigned to other.

Table S1. The threshold cycle (Ct) of *rpoD* copy number deposited on plants by Colorado potato beetle larvae. *rpoD* copy numbers were measured to estimate the amount of *Pseudomonas* sp. Deposited on leaves after 2 h of insect feeding. Values are untransformed means ± SEM (N = 5-6). Con, undamaged plants; AB(-), plants damaged by untreated larvae; AB(+), plants damaged by AB-treated larvae.

rpoD on potato leaves damaged by tomato fed larvae

Treatment	Ct*
Con	39.8 ± 0.2

rpoD on potato leaves damaged by potato fed larvae

Treatment	Ct
Con	39.7 ± 0.2

rpoD on buffalobur damaged by buffalobur fed larvae

Treatment	Ct
Con	39.5 ± 0.3
AB(-)	39.8 ± 0.2
AB(+)	39.7 ± 0.2

* Ct value of 40.0 represent that zero copy of *rpoD*.

Table S2. Alpha diversity indexes with singletons from Colorado potato beetle larvae that were reared on different host plants.

Host	Coverage	OTUs	Chao1	Shannon	1/Simpson
Tomato-1	0.996	74	77.5	2.88	9.38
Tomato-2	0.999	106	158.5	1.18	2.30
Tomato-3	1.000	67	75.5	1.51	3.79
(mean ± SEM)		(82.3 ± 12.0)	(103.8 ± 27.4)	(1.86 ± 0.52)	(5.16 ± 2.16)
Potato-1	1.000	60	126.4	0.67	1.50
Potato-2	0.999	61	115.1	0.82	1.85
Potato-3	1.000	39	59.0	0.32	1.15
(mean ± SEM)		(53.3 ± 7.2)	(100.2 ± 20.1)	(0.61 ± 0.15)	(1.50 ± 0.20)
Eggplant-1	0.999	57	71.0	0.17	1.06
Eggplant-2	1.000	38	59.1	0.66	1.80
Eggplant-3	1.000	26	39.0	0.55	1.53
(mean ± SEM)		(40.3 ± 9.0)	(56.37 ± 9.3)	(0.46 ± 0.15)	(1.46 ± 0.22)
Buffalobur-1	0.999	64	149.0	1.16	2.99
Buffalobur-2	0.999	72	222.5	1.08	2.64
Buffalobur-3	0.999	55	90.1	0.95	2.08
(mean ± SEM)		(63.7 ± 4.9)	(153.9 ± 38.3)	(1.06 ± 0.06)	(2.57 ± 0.26)
Horsenettle-1	1.000	21	32.0	0.26	1.13
Horsenettle-2	1.000	20	36.5	0.30	1.19
Horsenettle-3	1.000	16	25.3	0.20	1.10
(mean ± SEM)		(19.0 ± 1.5)	(31.3 ± 3.2)	(0.25 ± 0.03)	(1.14 ± 0.03)
Nightshade-1	1.000	51	59.3	0.93	1.70
Nightshade-2	1.000	30	36.9	0.16	1.06
Nightshade-3	0.999	58	125.7	0.58	1.54
(mean ± SEM)		(46.3 ± 8.4)	(73.9 ± 26.7)	(0.56 ± 0.22)	(1.44 ± 0.19)

Table S3. Relative abundance of the 10 most abundant OTUs from larvae that were reared on different host plants.

Host	Enterobacter OTU01	Lactococcus OTU02	Stenotrophomonas OTU03	Lactobacillus OTU05	Serratia OTU06	Enterococcus OTU08	Pseudomonas OTU09	Ralstonia OTU11	Sphingomonas OTU14	Spiroplasma OTU17
Tomato-1	1.51	0.11	16.92	-	4.00	1.66	0.68	12.32	6.46	22.48
Tomato-2	1.13	23.54	62.18	8.99	-	-	0.04	1.17	0.04	-
Tomato-3	26.03	36.83	8.84	23.16	0.19	-	3.78	0.30	0.04	-
(mean)	(9.56)	(20.16)	(29.32)	(10.72)	(1.40)	(0.55)	(1.50)	(4.60)	(2.18)	(7.49)
Potato-1	79.86	7.52	-	-	0.91	11.56	-	0.04	-	-
Potato-2	69.55	6.31	-	-	0.34	23.57	-	-	-	-
Potato-3	93.01	1.51	4.95	-	-	0.23	-	-	0.08	-
(mean)	(80.81)	(5.11)	(1.65)	-	(0.42)	(11.79)	-	(0.01)	(0.03)	-
Eggplant-1	2.38	96.83	0.08	-	0.11	-	-	0.11	-	-
Eggplant-2	34.04	65.62	-	-	0.08	-	-	0.11	-	-
Eggplant-3	20.21	79.68	0.04	-	-	-	-	-	0.04	-
(mean)	(18.88)	(80.71)	(0.04)	-	(0.06)	-	-	(0.08)	(0.01)	-
Buffalobur-1	33.93	24.59	-	40.46	0.08	0.08	-	0.08	-	0.53
Buffalobur-2	25.05	22.36	-	51.98	-	-	-	0.04	-	0.38
Buffalobur-3	9.48	24.29	-	64.71	0.76	0.19	-	0.08	-	0.23
(mean)	(22.82)	(23.75)	-	(52.39)	(0.28)	(0.09)	-	(0.06)	-	(0.38)
Horsenettle-1	4.19	94.41	-	0.04	1.28	-	-	-	-	-
Horsenettle-2	8.88	91.05	-	-	-	-	-	-	-	-
Horsenettle-3	4.00	95.92	-	-	-	-	-	-	-	-
(mean)	(5.69)	(93.79)	-	(0.01)	(0.43)	-	-	-	-	-
Nightshade-1	16.62	75.41	1.81	-	0.23	-	0.08	1.10	0.60	1.17
Nightshade-2	1.74	96.86	-	-	-	-	-	0.08	-	1.28
Nightshade-3	77.71	0.11	-	-	0.04	-	-	-	-	21.99
(mean)	(32.02)	(57.46)	(0.60)	-	(0.09)	-	(0.03)	(0.39)	(0.20)	(8.15)

Table S4. OTU table of bacterial communities from Colorado potato beetle larvae that were reared on different host plants. OTUs over 1% of total reads were included.

OTU ID	Domain	Phylum	Class	Order	Family	Genus
01	Bacteria	Proteobacteria	Gammaproteobacteria	Enterobacteriales	Enterobacteriaceae	Enterobacter
02	Bacteria	Firmicutes	Bacilli	Lactobacillales	Streptococcaceae	Lactococcus
03	Bacteria	Proteobacteria	Gammaproteobacteria	Xanthomonadales	Xanthomonadaceae	Stenotrophomonas
04	Bacteria	Proteobacteria	Gammaproteobacteria	Enterobacteriales	Enterobacteriaceae	unclassified
05	Bacteria	Firmicutes	Bacilli	Lactobacillales	Lactobacillaceae	Lactobacillus
06	Bacteria	Proteobacteria	Gammaproteobacteria	Enterobacteriales	Enterobacteriaceae	Serratia
08	Bacteria	Firmicutes	Bacilli	Lactobacillales	Enterococcaceae	Enterococcus
09	Bacteria	Proteobacteria	Gammaproteobacteria	Pseudomonadales	Pseudomonadaceae	Pseudomonas
11	Bacteria	Proteobacteria	Betaproteobacteria	Burkholderiales	Burkholderiaceae	Ralstonia
13	Bacteria	Proteobacteria	Gammaproteobacteria	Pseudomonadales	Moraxellaceae	Acinetobacter
14	Bacteria	Proteobacteria	Alphaproteobacteria	Sphingomonadales	Sphingomonadaceae	Sphingomonas
17	Bacteria	Tenericutes	Mollicutes	Entomoplasmatales	Spiroplasmataceae	Spiroplasma
18	Bacteria	Proteobacteria	Gammaproteobacteria	Pseudomonadales	Pseudomonadaceae	unclassified
21	Bacteria	Proteobacteria	Betaproteobacteria	Burkholderiales	Burkholderiaceae	Ralstonia
23	Bacteria	Proteobacteria	Betaproteobacteria	Burkholderiales	Burkholderiaceae	Burkholderia
28	Bacteria	Firmicutes	Bacilli	Bacillales	Staphylococcaceae	Staphylococcus
29	Bacteria	unclassified	unclassified	unclassified	unclassified	unclassified
30	Bacteria	Proteobacteria	Alphaproteobacteria	Rhizobiales	Bradyrhizobiaceae	Bradyrhizobium
36	Bacteria	unclassified	unclassified	unclassified	unclassified	unclassified
38	Bacteria	Proteobacteria	Alphaproteobacteria	Caulobacterales	Caulobacteraceae	Phenylobacterium
40	Bacteria	Proteobacteria	Gammaproteobacteria	Pseudomonadales	Pseudomonadaceae	unclassified
42	Bacteria	Proteobacteria	Betaproteobacteria	Burkholderiales	Comamonadaceae	Curvibacter
44	Bacteria	Proteobacteria	Alphaproteobacteria	Rhodobacterales	Rhodobacteraceae	Paracoccus
55	Bacteria	Proteobacteria	Betaproteobacteria	Neisseriales	Neisseriaceae	unclassified
60	Bacteria	Firmicutes	Bacilli	Lactobacillales	Streptococcaceae	Streptococcus
61	Bacteria	Proteobacteria	Alphaproteobacteria	Sphingomonadales	Sphingomonadaceae	Sphingomonas
62	Bacteria	Proteobacteria	Gammaproteobacteria	Enterobacteriales	Enterobacteriaceae	unclassified
103	Bacteria	Actinobacteria	Actinobacteria	Actinomycetales	Streptomycetaceae	unclassified
123	Bacteria	Tenericutes	Mollicutes	Entomoplasmatales	Spiroplasmataceae	Spiroplasma
134	Bacteria	Proteobacteria	Betaproteobacteria	Burkholderiales	Oxalobacteraceae	Undibacterium

138	Bacteria	Firmicutes	Bacilli	Lactobacillales	Streptococcaceae	Streptococcus
144	Bacteria	Actinobacteria	Actinobacteria	Actinomycetales	Dermabacteraceae	Brachybacterium
154	Bacteria	Bacteroidetes	Sphingobacteria	Sphingobacteriales	Chitinophagaceae	Hydrotalea
160	Bacteria	Proteobacteria	Gammaproteobacteria	Pseudomonadales	Moraxellaceae	Acinetobacter
169	Bacteria	Bacteroidetes	Sphingobacteria	Sphingobacteriales	Chitinophagaceae	unclassified
172	Bacteria	Bacteroidetes	unclassified	unclassified	unclassified	unclassified
175	Bacteria	Firmicutes	Bacilli	Bacillales	Bacillales_Incertae_Sedis_XI	Gemella
189	Bacteria	Acidobacteria	Acidobacteria	unclassified	unclassified	unclassified
223	Bacteria	Proteobacteria	Alphaproteobacteria	Rhizobiales	Phyllobacteriaceae	Phyllobacterium
232	Bacteria	Actinobacteria	Actinobacteria	Actinomycetales	Mycobacteriaceae	Mycobacterium
258	Bacteria	Firmicutes	Clostridia	Clostridiales	Ruminococcaceae	Ruminococcus
276	Bacteria	Proteobacteria	Gammaproteobacteria	Pasteurellales	Pasteurellaceae	Haemophilus
285	Bacteria	Firmicutes	Clostridia	Clostridiales	Lachnospiraceae	unclassified
293	Bacteria	Actinobacteria	Actinobacteria	Actinomycetales	Corynebacteriaceae	Corynebacterium
342	Bacteria	Proteobacteria	Alphaproteobacteria	Rhodospirillales	Acetobacteraceae	Acidocella
406	Bacteria	Actinobacteria	Actinobacteria	Actinomycetales	unclassified	unclassified
444	Bacteria	Acidobacteria	Acidobacteria	unclassified	unclassified	unclassified
448	Bacteria	Proteobacteria	Alphaproteobacteria	Caulobacterales	Caulobacteraceae	Brevundimonas
470	Bacteria	Firmicutes	Clostridia	Clostridiales	Lachnospiraceae	Clostridium
499	Bacteria	Bacteroidetes	Flavobacteria	Flavobacteriales	Flavobacteriaceae	unclassified
652	Bacteria	Proteobacteria	Betaproteobacteria	Neisseriales	Neisseriaceae	Neisseria
720	Bacteria	Proteobacteria	Deltaproteobacteria	Bdellovibrionales	Bdellovibrionaceae	Bdellovibrio
723	Bacteria	Firmicutes	Bacilli	Lactobacillales	Aerococcaceae	Abiotrophia
755	Bacteria	Bacteroidetes	Sphingobacteria	Sphingobacteriales	Chitinophagaceae	Sediminibacterium
1235	Bacteria	Firmicutes	Bacilli	Lactobacillales	Carnobacteriaceae	Dolosigranulum
1397	Bacteria	Acidobacteria	Acidobacteria	unclassified	unclassified	unclassified
1482	Bacteria	unclassified	unclassified	unclassified	unclassified	unclassified
1513	Bacteria	Firmicutes	Bacilli	Lactobacillales	Streptococcaceae	Lactococcus
1552	Bacteria	Bacteroidetes	Bacteroidia	Bacteroidales	Prevotellaceae	unclassified
1578	Bacteria	Firmicutes	Clostridia	Clostridiales	Lachnospiraceae	unclassified
1580	Bacteria	Actinobacteria	Actinobacteria	Actinomycetales	Corynebacteriaceae	Corynebacterium
